# A Sequential Sampling Approach to the Integration of Habits and Goals

**DOI:** 10.1007/s42113-024-00199-4

**Published:** 2024-03-05

**Authors:** Chao Zhang, Arlette van Wissen, Ron Dotsch, Daniël Lakens, Wijnand A. IJsselsteijn

**Affiliations:** 1 Human-Technology Interaction Group, Eindhoven University of Technology, PO Box 513, 5600 MB Eindhoven, The Netherlands; 2 Digital Engagement, Cognition and Behavior Group, Philips Research, Eindhoven, The Netherlands; 3 Present Address: Amsterdam, Netherlands

**Keywords:** Habit formation, Reinforcement learning, Habit-goal conflict, Sequential sampling models, Computational modeling, Decision field theory

## Abstract

Habits often conflict with goal-directed behaviors and this phenomenon continues to attract interests from neuroscientists, experimental psychologists, and applied health psychologists. Recent computational models explain habit-goal conflicts as the competitions between two learning systems, arbitrated by a central unit. Based on recent research that combined reinforcement learning and sequential sampling, we show that habit-goal conflicts can be more parsimoniously explained by a dynamic integration of habit and goal values in a sequential sampling model, without any arbitration. A computational model was developed by extending the multialternative decision field theory with the assumptions that habits bias starting points of preference accumulation, and that goal importance and goal relevance determine sampling probabilities of goal-related attributes. Simulation studies demonstrated our approach’s ability to qualitatively reproduce important empirical findings from three paradigms – classic devaluation, devaluation with a concurrent schedule, and reversal learning, and to predict gradual changes in decision times. In addition, a parameter recovery exercise using approximate Bayesian computation showcased the possibility of fitting the model to empirical data in future research. Implications of our work for habit theories and applications are discussed.

**Supplementary Information:**

The online version contains supplementary material available at 10.1007/s42113-024-00199-4.

## Introduction

Habits and routines make up a large part of motivated behaviors in humans and animals. While habits often serve goal-pursuits, psychologists have been fascinated by the situations where they conflict with each other. In daily life, people often repeat behaviors that benefited them in the past but compromise their current best interests. For example, at a road junction, a driver may quickly turn to the route that they usually take for years, despite being aware of an ongoing construction that blocks that road. In laboratory instrumental learning experiments, when humans and animals are extensively trained to behave in certain ways, their behaviors become insensitive to the devaluation of the original goals that motivate those behaviors (e.g., Adams, 1982; Dickinson, 1985; Tricomi et al., 2009; but see de Wit et al., 2018 for failed replications in humans). In social and health psychology, strong habits have been shown to attenuate the influences of goal-related constructs (i.e., attitude, intention) on health behaviors (e.g., Triandis, 1977; Verplanken et al., 1994; Zhang et al., 2022a, b; for reviews, see Gardner, 2015; Gardner et al., 2020). It is generally believed in psychology and neuroscience that goal-directed learning and habit learning are two distinct yet interacting systems in the brain (Daw, 2018; Dolan & Dayan, 2013; Wood et al., 2022; Yin & Knowlton, 2006), but the exact mechanism of their interaction remains an open and intriguing question.

**Fig. 1:**
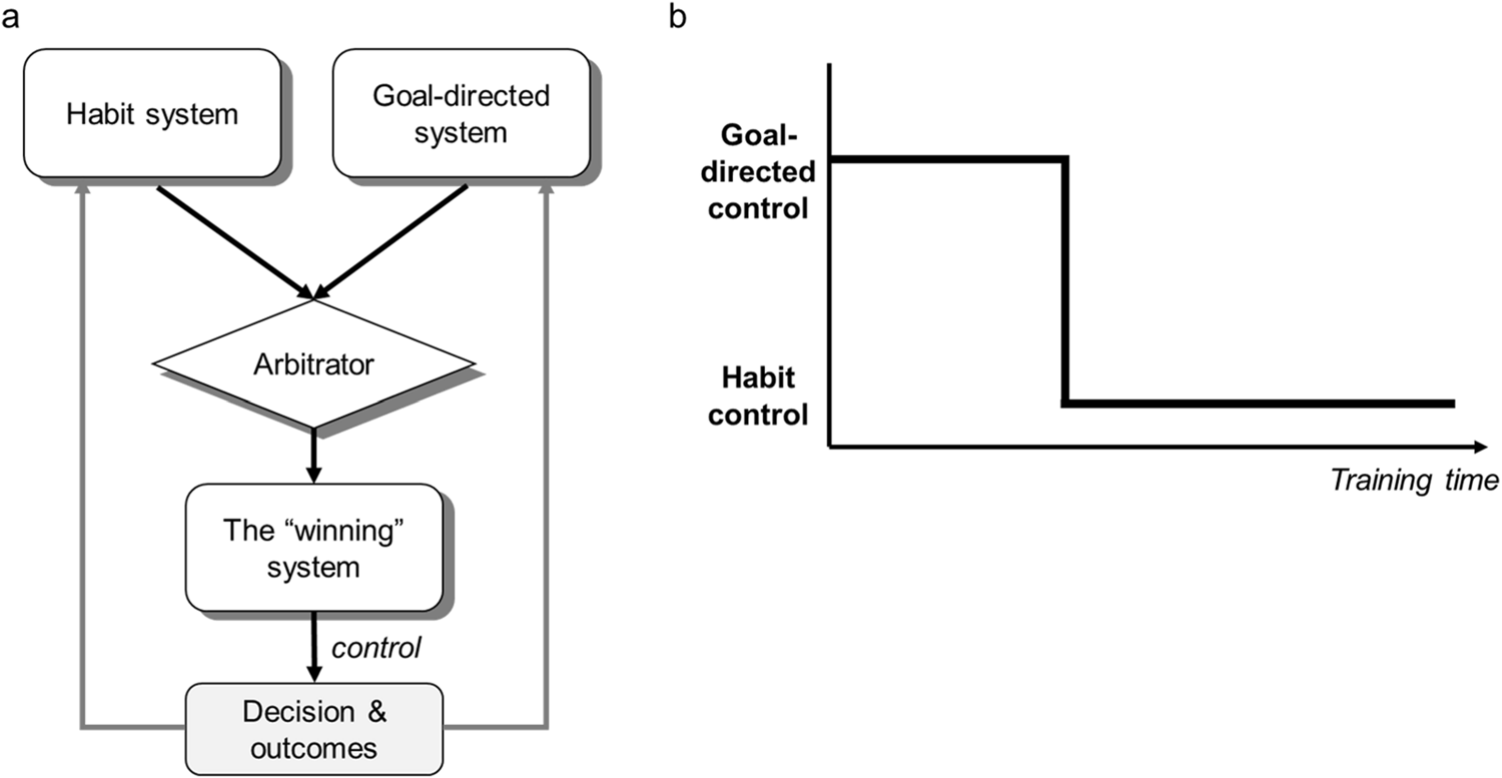
**a** A common scheme for arbitration models; **b** Predicted by arbitration models, control of response selection shifts from the goal-directed system to the habit system after a certain amount of training. These representations assume a “winner-takes-all” mechanism for response selection

A principal way to understand the functioning of cognitive systems is through computational modeling (Farrell & Lewandowsky, 2018). Following a general reinforcement learning framework, many researchers have attempted to model habit-goal interaction as a competition between two distinct learning systems (e.g., model-free and model-based reinforcement learning), arbitrated by a central control unit (e.g., Daw et al., 2005; Keramati et al., 2011; Miller et al., 2019; Pezzulo et al., 2013). Despite the differences among these models in terms of theoretical perspective and algorithmic implementation, arbitration models share the same conceptual scheme (Fig. 1a). In two distinct learning systems, action values of different behavioral responses are learned, representing how much these responses satisfy the current or past goals of a learning agent. Because action values learned in the two systems may be in disagreement, an arbitration or meta-choice process is needed to decide which system controls behavior based on the relative strengths of the two systems. For example, either the habit or the goal system takes control if that system estimates action values with less uncertainty (Daw et al., 2005) or maximizes the variance of action-outcome contingencies or habit strengths among different behavioral responses (Miller et al., 2019). Other models use the “cached” action values from the habit system by default, but switches to the action values updated by the goal-directed system when the arbitrator recognizes that the benefit of the switching (e.g., increased accuracy) exceed its cost (e.g., extra time spent on model-based tree search) (e.g., Keramati et al., 2011; Pezzulo et al., 2013; see also Kool et al., 2017). After arbitration, the probability of selecting each behavioral response is proportional to its final action value (i.e., passing through a softmax function). Because the habit system lags behind the goal system in reaching its maximum performance but is ultimately more efficient, the control of behavior shifts from the goal-directed system to the habit system in the later stage of learning (see Fig. 1b). Note that our discussion so far assumes a “winner-takes-all” mechanism, but the relative influences of the two systems on response selection can also be weighted based on the same arbitration rules (e.g., by their uncertainties) and the shift from goal-directed control to habit control will then be gradual.

Arbitration models have been successful in qualitatively reproducing some classic empirical findings in the instrumental learning literature, such as the insensitivity to goal devaluation effect (Daw et al., 2005; Keramati et al., 2011; Miller et al., 2019), and new predictions from the models were supported by results from sequential decision experiments (e.g., Kool et al., 2017). Despite this success, arbitration models are not without problems. While the two separate learning systems and their neurological substrates are well-established (Yin & Knowlton, 2006), the existence of an additional arbitrator in the brain remains a critical assumption, awaiting more neurophysiological evidence (but see Lee et al., 2014). Moreover, compared to the sophisticated reinforcement learning algorithms used for habit and goal-directed learning, response selection in all previous models is simplified as a softmax function. In other words, the response selection process is an “empty” model, with no cognitive process nor mechanism specified (Pedersen et al., 2017). This creates two further problems. First, after arbitration, the response selection process is the same, regardless of which system is in control. This contradicts with the seemingly qualitative differences in how habits and goals influence behaviors – habitual responses are often conceptualized as impulses triggered by environmental cues (see Wood & Neal, 2007), which are sometimes overruled by goals. Second, the lack of a process model for response selection makes arbitration models ill-suited for accounting for the change of decision time over the course of habitualization. Some arbitration models imply identical decision times for responses controlled by habits and goals (e.g., Daw et al., 2005; Miller et al., 2019), while other models produce unrealistic sudden switches between very fast (habitual) and very slow (goal-directed) responses (e.g., Keramati et al., 2011).

Very recently, there is a growing interest in using sequential sampling models as the response selection module in reinforcement learning models (Dunovan & Verstynen, 2016; Fontanesi et al., 2019; Frank et al., 2015; Miletić et al., 2020; Pedersen et al., 2017). In decision-making research, sequential sampling (also known as evidence accumulation) refers to a class of dynamic models implementing a “race-to-threshold” mechanism (for reviews, see Forstmann et al., 2016; Oppenheimer & Kelso, 2015), including drift diffusion models (e.g., Ratcliff, 1978; Ratcliff & Rouder, 1998), the linear ballistic accumulator model (e.g., Trueblood et al., 2014), and decision field theories (e.g., Busemeyer & Townsend, 1993; Roe et al., 2001). All these models assume that a decision-maker accumulates evidence or preferences for different response options by sampling information from their environment and/or memory, and once the accumulated evidence or preference for a certain response option exceeds a decision threshold, the final decision is made. Within a sequential sampling framework, the learned action values from reinforcement learning algorithms can be mapped to the speeds of accumulation for different response options (i.e., drift rates), instead of being fed to a softmax function. Recent empirical studies have shown that models combining reinforcement learning and drift diffusion models can adequately account for both choice and decision time data obtained from human instrumental learning experiments (Fontanesi et al., 2019; Frank et al., 2015; Pedersen et al., 2017). Given these results and the success of sequential sampling models in many other areas (Forstmann et al., 2016), we hypothesized that a sequential sampling approach can also be used to explain habit-goal interactions, if habits and goals can be mapped to distinct parameters in a sequential sampling model.

Two distinct determinants of any sequential sampling process are the *starting point* of evidence or preference accumulation (baseline evidence strength or preference) and the *drift rate* at each step of accumulation (Forstmann et al., 2016). When introducing the multialternative decision field theory (MDFT), Roe et al. (2001) discussed a possible mapping of habits and goals to starting point and drift rate respectively, but the idea was not examined any further in the context of value-based decision-making. It is now customized to assume that the goal-directed system influences the sequential sampling process by changing the drift rates of different response options based on the action-values learned from reinforcement learning (Fontanesi et al., 2019; Frank et al., 2015; Pedersen et al., 2017), but the mapping between habit strength and starting point remains unexplored. Empirical evidence for the latter mapping comes mainly indirectly from perceptual decision-making research, where a typical task requires judging the movement direction of groups of dots. It was found that while drift rate related to stimulus ambiguity in the current trial, starting point related instead to past choices (Bode et al., 2012; Mulder et al., 2012; van Ravenzwaaij et al., 2012; but see Urai et al., 2019). If a similar distinction between past and current information applies to value-based decision-making, then habits and goal-related action values may play the same roles as past choices and current perceptual evidence respectively. Furthermore, Akaishi and colleagues (Akaishi et al., 2014) found that the way past choices influence current choice in the perceptual domain is mathematically equivalent to a form of Hebbian learning (Hebb, 1949), which has been previously theorized to also underlie habit learning (Klein et al., 2011; Miller et al., 2019). Finally, the idea of having different starting points for different response options is mathematically equivalent to an idea that some response options start the accumulation process earlier in time or certain options gain some preferences in a separate initial stage of accumulation. The latter idea has been explored in a two-stage drift diffusion model where sampling from memory precedes a second stage of sampling from perceptual information (Bornstein et al., 2018; Wang et al., 2022). In a more general sense, elevated starting points can be understood as stronger baseline preferences or a form of early preparation for the habitual response (see Hardwick et al., 2019).

In this paper, we formally propose a sequential sampling model in which habits and goals are integrated dynamically and examine whether our model can qualitatively reproduce some well-known empirical demonstrations of habit-goal conflicts that were previously explained by arbitration models. We argue that a successful application of sequential sampling to habit-goal interaction can make three theoretical contributions. First, by mapping habits and goals directly to parameters in a sequential sampling model, our new approach does not require an arbitration between two learning systems and thus circumvent the need of finding an “arbitrator” in the brain. Of course, we do not rule out the possibility that some arbitration-like processes are functionally useful and neurobiologically plausible, but as long as there is no strong evidence, sequential sampling provides a neurobiologically-plausible alternative (see Busemeyer et al., 2019; Dunovan & Verstynen, 2016). Second, the sequential sampling approach offers a principal way of explaining both decisions (behavioral responses) and decision time over the course of learning and habitualization. Conceptually, one can easily expect that as strong habits lead to starting points closer to the decision threshold, it would take less time to make a decision (i.e., reaching the threshold) and leave less opportunities for the goal-directed system to influence the accumulation process. Finally and more broadly, adding to previous works (Dunovan & Verstynen, 2016; Fontanesi et al., 2019; Frank et al., 2015; Miletić et al., 2020; Pedersen et al., 2017), a useful model that combines reinforcement learning and sequential sampling contributes to a more unified approach for modeling learning and decision-making in humans and other organisms.

In the remainder of the paper, we first present our computational model that extends the MDFT by adding a goal-directed and a habit learning component. Next, in three simulation studies, we show that the proposed model can reproduce choice and decision time patterns found in three instrumental learning tasks – classic devaluation paradigm, devaluation paradigm with a concurrent schedule, and reversal learning, which were all used previously to validate the arbitration models. Furthermore, in order to evaluate the possibility of estimating model parameters from data, we report the results of a small-scale parameter recovery exercise.^1^ Finally, implications of the findings for habit research and value-based decision-making are discussed, as well as limitations and suggestions for future work.

## The Conceptual and the Computational Model

**Fig. 2:**
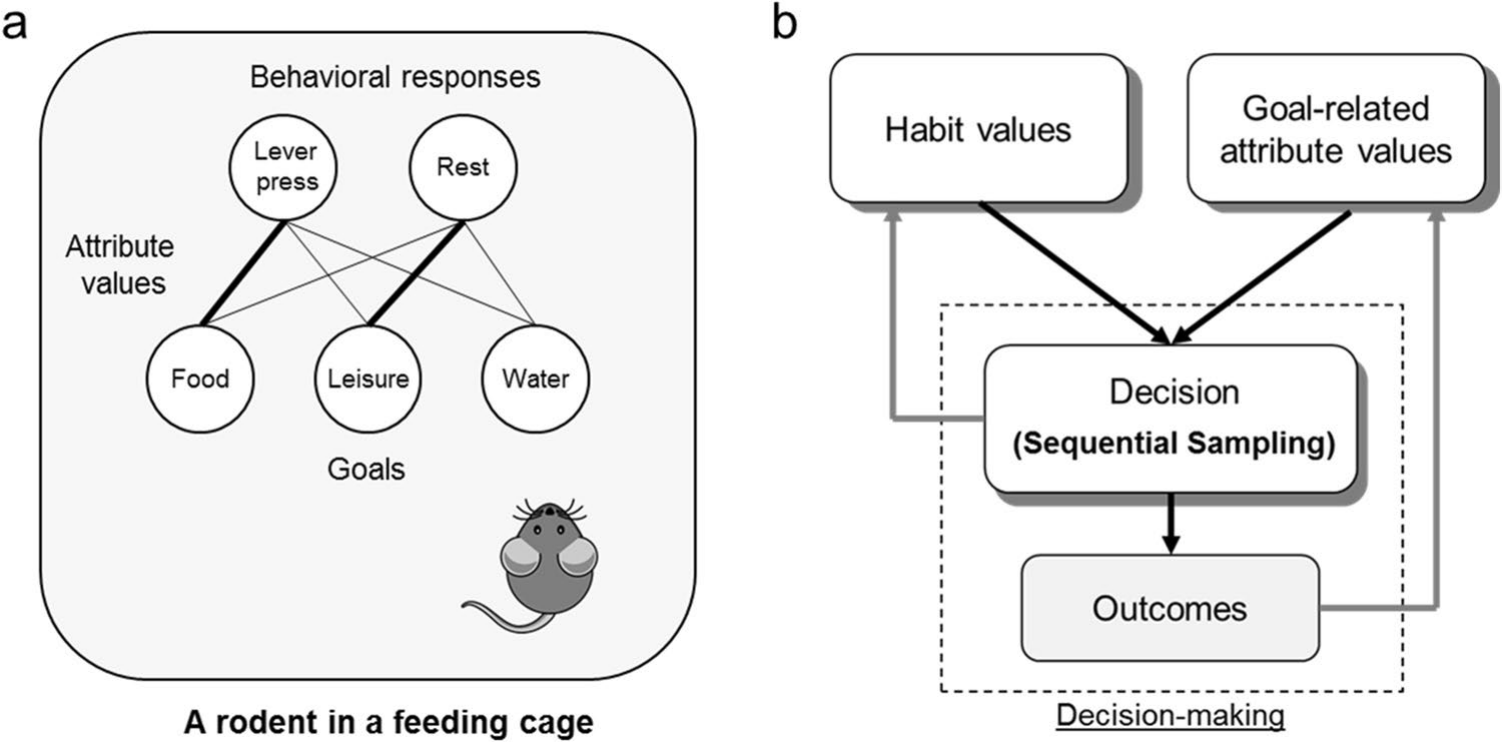
**a** A representation of behavioral responses, goals, and goal-related attribute values (thicker lines represent higher values) in a typical instrumental learning experiment with rodents; **b** A representation of the task as repeated alternations between decision-making and learning

We first defined the structure of a typical instrumental learning task using an example of rodents learning to press a lever to obtain food (Fig. 2a), but the same task definition also applies to humans. In a constrained environment (e.g., a feeding cage), a learning agent is assumed to have a fixed number of goals that differ in their importance or *goal values*. For example, a rodent may strive primarily to obtain food, water, and mating opportunities, but sometimes also to enjoy leisure. To satisfy its goals, the agent needs to engage in certain behaviors, and it can be assumed that given the constrained environment, only a limited number of behavioral responses are available, for example, to press a lever or to rest. For each goal-response pair, an *attribute value* represents the likelihood of achieving the goal by executing the behavior (e.g., lever-pressing scores high on attribute *food*, resting scores high on attribute *leisure*). Note that among all the goal-related attributes, some can be called *unattainable attribute*s as no behavioral response in the constrained environment satisfies the associated goals (e.g., *mating* is an unattainable attribute given no other rodents in the cage). Finally, unrelated to goals,^2^ each behavioral response also holds a habit value, depending on how frequently the response was selected in the same task environment in the past (Thorndike, 1932). Cognitively, habit values reflect the strengths of mental associations between behaviors and environmental cues (Wood & Neal, 2007; Wood & Rünger, 2016).

Overall, the task of a learning agent is to search for the behavioral response that maximizes the satisfaction of its various goals through repeated decisions. This representation is similar to the multi-armed bandit task in the reinforcement learning literature, where an agent learns the pay-offs of multiple slot machines through repeated decision trials (Sutton & Barto, 1998; for a similar representation of instrumental learning, see Fontanesi et al., 2019). Conceptually, the learning task consists of a sequence of interconnected decision-making (response-selection) and learning processes (Fig. 2b). At each iteration, the current goal values, attribute values, and habit values are integrated in a sequential sampling process to produce a decision (e.g., press the lever) and its associated outcomes (e.g., food delivered). Following the decision, perceived outcomes are used to update the agent’s beliefs about the attribute values of the behavioral responses (*goal-directed learning*). Also, the habit values of the behavioral responses are updated based simply on whether the responses are selected at this iteration (*habit learning*). The updated attribute values and habit values are used for the subsequent decisions.

### Modeling Response Selection as a Sequential Sampling Process

**Fig. 3:**
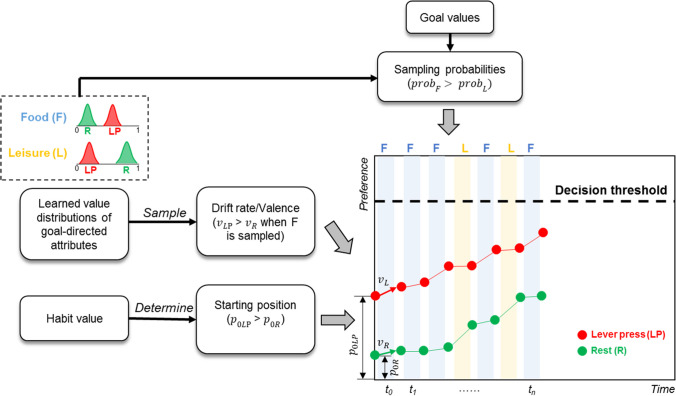
A conceptual representation of a sequential sampling process and its inputs

For modeling response selection in the instrumental learning task, we adopted the general framework of the MDFT (Roe et al., 2001), but other sequential sampling models of value-based decision-making should also work in principle (e.g., Trueblood et al., 2014; Usher & McClelland, 2001). Figure 3 illustrates the model conceptually, showing how the outcome and time course of a response selection are determined in a sequential sampling process as influenced by four variables – *starting points*, *sampling probabilities*, *drift rates* at each time step, and a *decision threshold.*^3^

At the start of a sequential sampling process, starting points represent a learning agent’s baseline preference towards a set of behavioral responses. The model proposes that habitual responses are by default more favorable than the less habitual ones, represented by higher starting points^4^ (Roe et al., 2001). The starting points or the preferences at *t*_*0*_ for all responses equal to their habit values (H) scaled by a scalar parameter $\theta$:

1 $${\mathbf{P}}_{\left(0\right)}= \theta \mathbf{H}$$

From their starting points, the agent’s preferences for different responses drift over time, and at each time step, the drifts depend on which goal-related attribute is sampled and how each response scores on the sampled attribute. For example, if attribute *food* is sampled, the preference for the response lever-press will increase greatly because lever-press scores high on attribute *food*. A key assumption of MDFT is that at one time step, the agent only samples one attribute, for example, either *food* or *leisure*. In the original MDFT, the sampling probabilities are equal for all attributes (i.e., sampling randomly). Instead, our model proposes that sampling probabilities of attributes are determined by two variables – the *goal values* of the attributes and the *attainability of attributes*, which measure the importance and relevance of the attributes respectively in the current task. If, for example, obtaining food is more important than conserving energy for rodents, *food* will be sampled more than *leisure*. Also, if one attribute is more attainable in the current task (contained more in the responses) than another attribute (e.g., some behavioral responses result in *food*, but none results in *mating*), it will be more likely to be sampled. Mathematically, a softmax function is used to calculate sampling probability (${Pr}_{j}$), with the multiplications of goal value (${G}_{j}$) and the attainability of attributes (${A}_{j}$) as inputs and $\tau$ as a scaling parameter,

2 $${Pr}_{j}= \frac{{e}^{\tau {G}_{j}{A}_{j}}}{{\sum }_{k=1}^{K}{e}^{\tau {G}_{k}{A}_{k}}}$$

where the attainability of each attribute is the sum of all responses’ scores on that attribute $({X}_{ij})$, ${A}_{j}= {\sum }_{i=1}^{N}{X}_{ij}$. Attribute values are often given externally in choice experiments, but in our learning task they are derived from learned probability distributions for each attribute. For the calculation of ${A}_{j}$, the model assumes that the expected mean reward values (EMRs) of the distributions are used. Later, attribute values sampled at each time step $({M}_{ij})$ are instead randomly sampled from the distributions.

Two implications of Eq. 2 are worth noting. First, the unattainable attributes will have very low though non-zero sampling probabilities. As there can be many unattainable attributes in a constrained task environment, the sum probability of sampling any unattainable attribute can be non-trivial, and it is similar to the probability of sampling noise, which is usually arbitrarily defined in sequential sampling models (e.g., Roe et al., 2001). Second, goal values for different attributes are assumed to be stable in short time frames for each agent, but can be substantially changed through experimental procedures such as goal devaluation (e.g., Adams, 1982; Dickinson, 1985). Consequently, if a food is devalued, its sampling probability decreases towards zero.

The rest of the model follows MDFT closely. When an attribute is selected based on sampling probabilities at time *t*, the momentary drift rates of behavioral responses (or *valences* as in Roe et al., 2001) are their attribute values on the sampled attribute, as in the matrix form:^5^

3 $${\mathbf{V}}_{\left(t\right)}={\mathbf{M}}_{\left(t\right)}{\mathbf{W}}_{\left(t\right)}$$

where ${\mathbf{V}}_{\left(t\right)}$ is an *N*-dimensional valence vector representing the drift rates of different behavioral responses at different time steps. ${\mathbf{W}}_{\left(t\right)}$is a *J*-dimensional vector of attribute weights, in which the sampled attribute is weighed 1 and all others are weighed 0. Lastly, ${\mathbf{M}}_{\left(t\right)}$ is an *N*-by-*J* matrix containing all attribute values for all responses. Unlike the original MDFT, where ${\mathbf{M}}_{\left(t\right)}$ is fixed at all *t*, ${\mathbf{M}}_{\left(t\right)}$elements are randomly sampled according to the underlying probability distribution learned for each response-attribute pair at each time step.

Next, preferences ${\mathbf{P}}_{\left(t\right)}$ at time *t* are determined by the preferences at the previous time step (${\mathbf{P}}_{(t-1)}$) and the current drift rates ${\mathbf{V}}_{\left(t\right)}$. Between two successive time steps, there is a decay or leakage of each preference itself, and there are influences from the preferences of competing responses in the form of lateral inhibition. Both processes are summarized in an *N*-by-*N* matrix **S**, in which elements on the main diagonal are equal to a self-decay parameter (${S}_{self}$) and all other elements are equal to a lateral inhibition parameter $({S}_{lateral})$. Thus, preferences are calculated in the matrix form:

4 $${\mathbf{P}}_{\left(t\right)}=\mathbf{S}{\mathbf{P}}_{(t-1)}+ {\mathbf{V}}_{\left(t\right)}$$

When a behavioral response’s preference exceeds the decision threshold, a decision is made and the behavior is executed by the learning agent. Reward to be received relating to each attribute or goal is calculated by reward probabilities pre-defined by the learning task (e.g., the reinforcement schedule of a learning experiment). Before making the next decision, habit values and goal-related attribute value distributions are updated.

### Modeling Habit Learning

We assumed that habits are value-free, meaning that their updates depend only on the decisions themselves but not on the consequences brought by the decisions. Specifically, the model for habit learning uses the same Hebbian learning equation as in Miller et al. (2019), but is also conceptually compatible with other equations (Klein et al., 2011; Psarra, 2016; Tobias, 2009):

5 $${\mathbf{H}}_{\left(T\right)}= {\mathbf{H}}_{(T-1)}+ {\alpha }_{H}({\mathbf{A}}_{H}- {\mathbf{H}}_{\left(T-1\right)})$$

where learning rate ${\alpha }_{H}$ controls how much habit values ($\mathbf{H}$) change from one time point to the next,^6^ and ${\mathbf{A}}_{H}$ is a scaling parameter which limits the upper-bound of habit values. The equation implies that with repeated behaviors, habit values increase fast at the beginning and then their growth slow down until the values reach their asymptotes. This pattern is consistent with empirical data on the dynamics of self-reported habit strength (Lally et al., 2010).

#### Modeling Goal-Directed Learning

Previous models have implemented model-based reinforcement learning algorithms for goal-directed learning (Daw et al., 2005; Keramati et al., 2011; Miller et al., 2019). Since we simplified our task representation to a single-state repeated decision-making or multi-armed bandit problem rather than a Markov decision process, goal-directed learning is modeled with a simple algorithm of Bayesian belief update – combining prior distributions (beliefs about attribute values before a decision) and data (perceived rewards) to obtain posterior distributions (beliefs after a decision). Assuming that the reward generation processes in learning experiments are Bernoulli processes, beta distributions can be used for both priors and posteriors. Formally, the updating rule is expressed as:

6 $$\left(\alpha_{ij},\beta_{ij}\right)\leftarrow\left\{\begin{array}{lc}\left(\left(1-\gamma\right)\alpha_{ij}+\gamma\overline\alpha,\left(1-\gamma\right)\beta_{ij}+\gamma\overline\beta\right),&D_{\left(T\right)}\neq i\\\left(\left(1-\gamma\right)\alpha_{ij}+\gamma\overline\alpha+R_{j\left(T\right)},\left(1-\gamma\right)\beta_{ij}+\gamma\overline\beta+1-R_{j\left(T\right)}\right),&D_{\left(T\right)}=i\end{array}\right.$$

where the alpha and beta parameters defining the beta distribution of response *i* on attribute *j* are only updated by reward ${R}_{j\left(T\right)}$, if decision at *T* (${D}_{\left(T\right)}$) is to choose response *i*. To account for the nonstationary environments in typical experimental setups (e.g., reward functions can be suddenly changed by the experimenter), parameter $\gamma$ is used to inject uncertainty in the distributions. In other words, belief distributions always regress to a default distribution defined by $\overline{\alpha }$ and $\overline{\beta }$ (a uniform beta distributions with both equaling 1), ensuring fast reactions of learning agents to changes in the environment.

## Simulation Studies

In all three simulation studies to be discussed, except for task-specific variables, the same parameter values were used for all model parameters introduced in the last section (see Table 1).

**Table 1:** Parameter values used in all three studies

	Parameter	Explanation	Value
Decision-making (Sequential sampling)	$\theta$	Scaling parameter for transforming habit strengths to starting points. The exact value is arbitrary, but it should scale the largest habit strength possible (close to 1) to the decision threshold (e.g., 1).	1
	$\tau$	Scaling parameter for the softmax function used in Eq. 2. The larger the value, the more dominant the largest input is in calculating the outputs. The value is arbitrary, but depends on the scale used for goal values, e.g., [0, 1]).	10
	$${S}_{self}$$	Leakage parameter that measures on the information loss ($$1- {S}_{self})$$in preference accumulation (e.g., 0.94 used in Roe et al., 2001).	0.99
	${S}_{lateral}$	Lateral inhibition parameter that measures the competition among behavioral responses (e.g., -0.001 and − 0.025 used in Roe et al., 2001).	-0.03
	$DT$	Decision threshold for sequential sampling. The exact value is arbitrary, as it depends on the scales used for attribute values (e.g., [0, 1] in our studies).	1
	$maxStep$	The maximum time step allowed in a sequential sampling process if no response’s preference exceeds decision threshold.	100
	${N}_{unattain}$	Number of unattainable attributes.	10
Habit learning	${\alpha }_{H}$	Learning rate in the Hebbian equation for habit learning. The larger its value, the faster habit strengths update. Miller et al. (2019) used much smaller values (e.g., 0.001), and indeed many more training trials were required to reach full habit strengths (e.g., 6000).	0.04
	${\mathrm{A}}_{H}$	Scaling parameter determining the upper bound of habit strength (usually 1, Miller et al., 2019).	1
Goal-directed learning	$\gamma$	Uncertainty parameter that determines the rate of uncertainty injected in the Bayesian belief updates. The larger its value, the faster a learner discounts “old” information, or “forgets” faster (e.g., 0.01 used in Russo et al., 2018).	0.1
	$\overline{\alpha }$	Alpha parameter of the convergence distribution in the absence of observations (uniform beta distribution was used, see Russo et al., 2018).	1
	$\overline{\beta }$	Beta parameter of the convergence distribution in the absence of observations.	1

### Study 1: Classic Devaluation Effect

The classic devaluation effect shows that learning agents become insensitive to goal devaluation after extensive training, but remain sensitive after moderate training. The effect has been repeatedly replicated for both animals and humans (e.g., Adams, 1982; Dickinson, 1985; Killcross & Coutureau, 2003; Liljeholm et al., 2015; Tricomi et al., 2009; Yin et al., 2004; Yin et al., 2005), and it has been considered a seminal finding for differentiating habits from goal-directed behaviors. The ability of reproducing the effect was also treated as a key empirical validation for the arbitration models (Daw et al., 2005; Keramati et al., 2011; Miller et al., 2019).

In a typical animal devaluation experiment, rodents learn to press a lever to obtain food pallets through either moderate or extensive pairing of the response and the food. After training, half of the rodents are subjected to a devaluation procedure, where the food becomes undesirable because of either a satiation procedure or a food-aversive conditioning (indicated as the “devalued” or “paired” group). The other half undertakes a similar procedure but with a different food not used in training (indicated as the “non-devalued” or “control” group). Finally, in an extinction test, no food pallets are delivered no matter how frequently the rodents press the lever. The devaluation effect manifests as an interaction effect. After moderate training, rodents in the devalued group press the lever less often than their peers in the control group. For rodents that receive extensive training, their lever-pressing responses seem to become insensitive to goal devaluation – both the devalued and the control group press the lever with equal frequency.

In the simulated experiment, learning agents were trained to press the lever for either 40 or 240 trials (as in Keramati et al., 2011), in which they were assumed to have a higher goal value for obtaining food (${G}_{food}=0.8$) than for having some rest (${G}_{leisure}=0.4$). Pressing the lever would lead to food 60% of the time,^7^ but never any leisure. Relaxing (no lever-pressing), on the other hand, always led to leisure but no food. Besides food and leisure, the agents were assumed to have 10 other important goals ${ (G}_{unattain}=0.8)$, but these goals were unattainable by either of the two responses. Devaluation was implemented as the diminishing of ${G}_{food}$ to 0 for half of the agents. In the 100 extinction trials, the probability of obtaining food by lever-pressing was reduced from 0.6 to 0. Five-hundred simulations of homogenous agents were run.

**Fig. 4:**
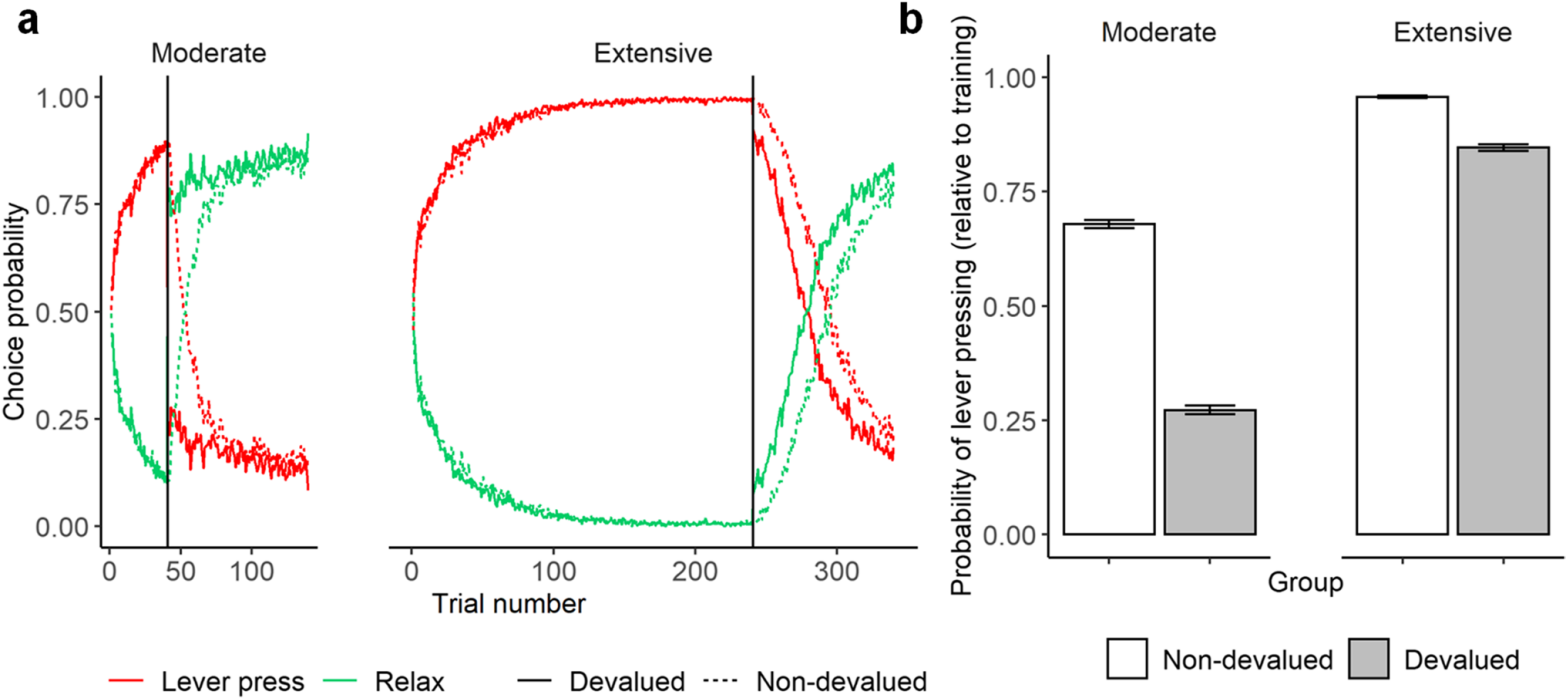
Simulated behavioral results for a classic devaluation experiment. **a** Change of choice probability over time; **b** Aggregated lever-pressing rates in the first 20 trials after devaluation relative to the level at the end of training

Figure 4 shows simulated choice probabilities over time and aggregated response rates. Our model produced a main effect of training (higher lever-pressing rates after extensive training), a main effect of devaluation (lower lever-pressing rates when ${G}_{food}$ is devalued), and most importantly a clear *training duratio*n by *devaluation* interaction effect. As can be seen in Fig. 4a, the lever-pressing rates in the two groups decreased almost in parallel after extensive training, while after moderate training the lever-pressing rate of the devalued group declined sharply as compared to the non-devalued group. Note that it was almost always the case that lever-pressing rate in the devalued group was slightly lower than in the control group (Fig. 4b), while in empirical studies equal rates in both groups or even slightly higher rate in the devalued group have been found (e.g., Dickinson, 1985). But this particular empirical pattern has also not been shown by the arbitration models (Daw et al., 2005; Keramati et al., 2011; Miller et al., 2019): they mainly compared response rates before and after devaluation, but not the relative response rates in the devalued and control groups after devaluation as usually reported in the empirical studies.

**Fig. 5:**
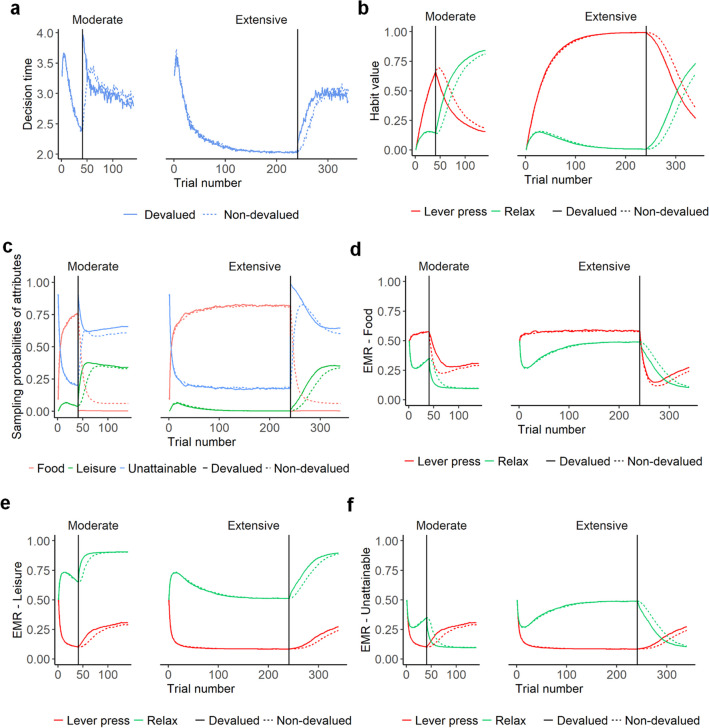
Temporal changes of decision times and underlying cognitive variables in the simulated devaluation experiment. **a** Decision time; **b** Habit value; **c** Sampling probability of attributes; **d** EMR of attribute *food*’s distributions; **e** EMR of attribute *leisure*’s distributions; **f** EMR of unattainable attributes’ distributions

Our model also predicted that decision times decreased gradually over the course of training, but increased abruptly after devaluation, before eventually decreasing again (see Fig. 5a). A notable novel prediction was an increase of decision times after devaluation was observed in all conditions, regardless of whether strong habits were formed or not (cf. Keramati et al., 2011).

The effect-generating mechanisms of the model are reflected in the temporal changes of the underlying cognitive variables in the model, especially at the transition from training to extinction (point of devaluation for the devalued group). First, as expected, the habit values for the two groups after extensive training were very close to 1, while the habit values after moderate training were just below 0.75 (Fig. 5b). Second, there was a sudden change in sampling probabilities for the devalued group – these agents stopped to sample attribute *food* because of the goal devaluation, but instead started to sample the unattainable attributes a lot (Fig. 5c, left). In contrast, agents in the control group continued to sample *food* frequently before they gradually unlearned the association between lever-pressing and food in the extinction phase (Fig. 5c, right). Thus, when looking at the expected mean reward values (EMR) for attribute *food* and the unattainable attributes (Fig. 5d & f), it was clear that the response lever-pressing was at disadvantage in the devalued group compared to the control group. The lever-pressing rate of the devalued group dropped significantly faster (Fig. 4a, left), unless the high habit values for the agents after extensive training functioned as a counteracting mechanism. 

**Fig. 6:**
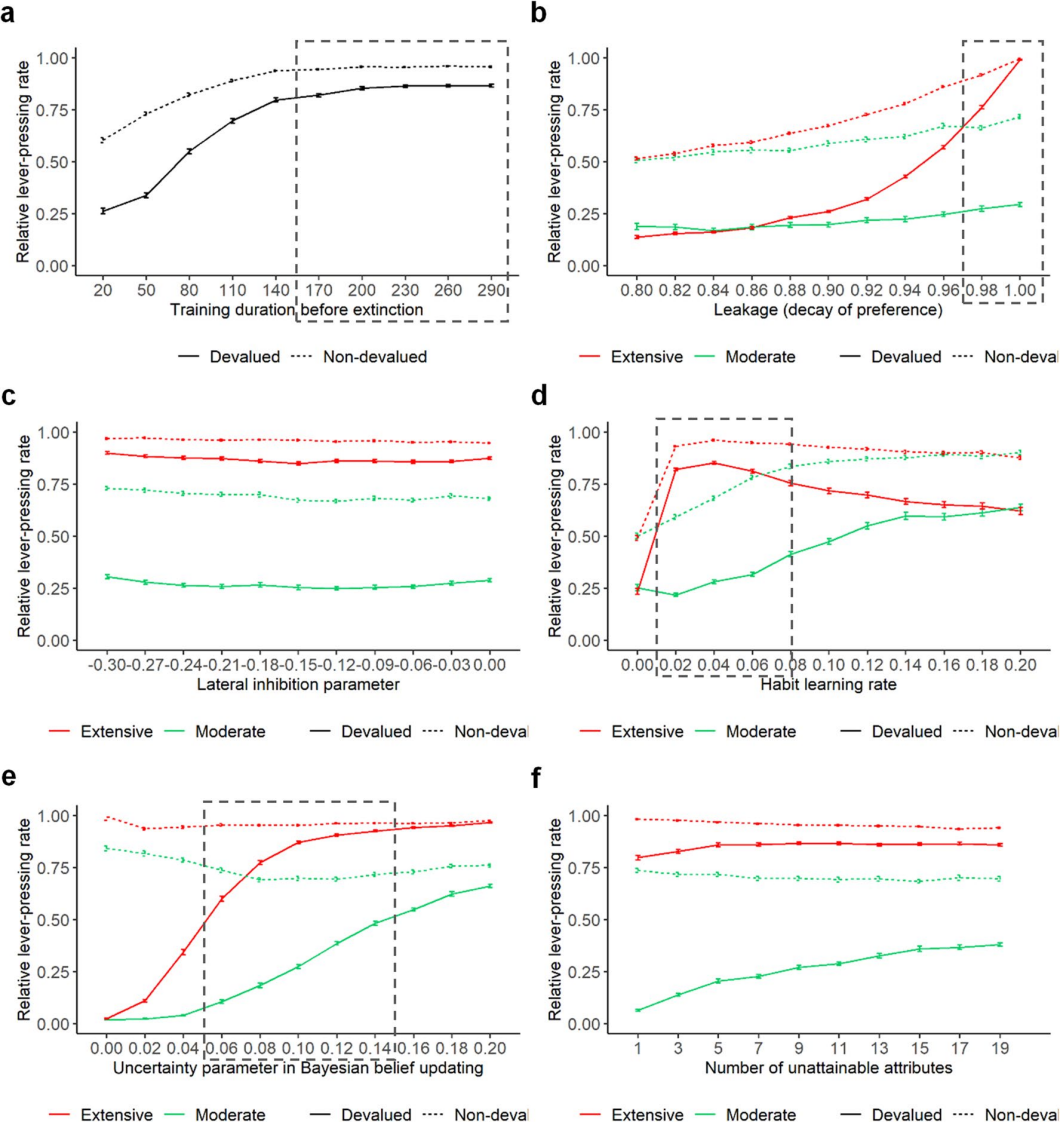
Sensitivity of the devaluation effect to different parameter values. **a** Training duration; **b** Leakage parameter $\left({S}_{self}\right)$; **c** Lateral inhibition parameter (${S}_{lateral}$); **d** Habit learning rate (${\alpha }_{H}$); **e** Uncertainty parameter in Bayesian belief updating ($\gamma$); **f** Number of unattainable attributes $({N}_{unattin})$. The dashed squares indicate the effect-producing ranges

Sensitivity analyses showed that the model was reasonably robust in reproducing the devaluation effect against changes in parameter values. First, as expected, lever-pressing rates in the devalued and control group only became comparable when the training was more than approximately 170 trials (Fig. 6a). This result reaffirmed the devaluation effect that insensitivity to goal devaluation only happens when the response is overtrained. Second, a very high value for the memory parameter (${S}_{self}$) was needed to reproduce the devaluation effect (Fig. 6b), consistent with the small memory leakages implemented in sequential sampling models in the literature (e.g., Roe et al., 2001). Third, the lateral inhibition parameter (${S}_{lateral}$) in the range of -0.3 and 0 did not change simulation results to any extent (Fig. 6c), and the relative low values used were consistent with the literature (e.g., Roe et al., 2001). Since theoretically lateral inhibition has an effect of reinforcing the responses with default high preferences (due to strong habits), a very large ${S}_{lateral}$ would result in an unrealistic pattern of no decay of lever-pressing rate in the extinction phase.

Fourth, the curves for habit learning rate confirmed that some habit formation was needed to reproduce the devaluation effect, but if habits were made to form too fast (e.g., ${\alpha }_{H}$ > 0.15), responses would become insensitive to goal devaluation even after moderate training (Fig. 6d). Fifth, results of the gamma parameter suggested that a small uncertainty injection was needed to reproduce the devaluation effect (Fig. 6e), as the parameter positively related to the value distributions of the unattainable attributes that were mostly sampled for the devaluated groups. If there was little uncertainty (e.g., $\gamma$ < 0.05), the resultant low value distributions would lead to drift rates that were too small to push the baseline preference of lever-pressing to the decision threshold even after extensive training. In contrast, if a lot of uncertainty was injected (e.g., $\gamma$ > 0.15), very large drift rates would be sampled from the value distributions of unattainable attributes and they would push baseline preferences of lever-pressing after both moderate and extensive training to the decision threshold. Finally, the number of unattainable attributes did not seem to have any substantial impact on the generation of the devaluation effect (Fig. 6f).

### Study 2: Devaluation Paradigm with a Concurrent Schedule

We extended our simulation to devaluation experiments with a concurrent schedule. In Kosaki and Dickinson (2010), instead of training one response-outcome pair, rodents were trained to learn two instrumental responses with two types of food concurrently. With this schedule, even if extensive training was used, rodents remained sensitive as to which food was devalued. Thus, we simulated 500 homogeneous agents only in extensive training to see if the model would produce a clear difference between responses to the devalued and non-devalued food. Other setups were similar to the previous scenario, except that two food attributes (with goal values ${G}_{food\_A}= {G}_{food\_B}=0.8$) and two lever-pressing responses were used. Each food was again reinforced to the correct response 60% of the time.

**Fig. 7:**
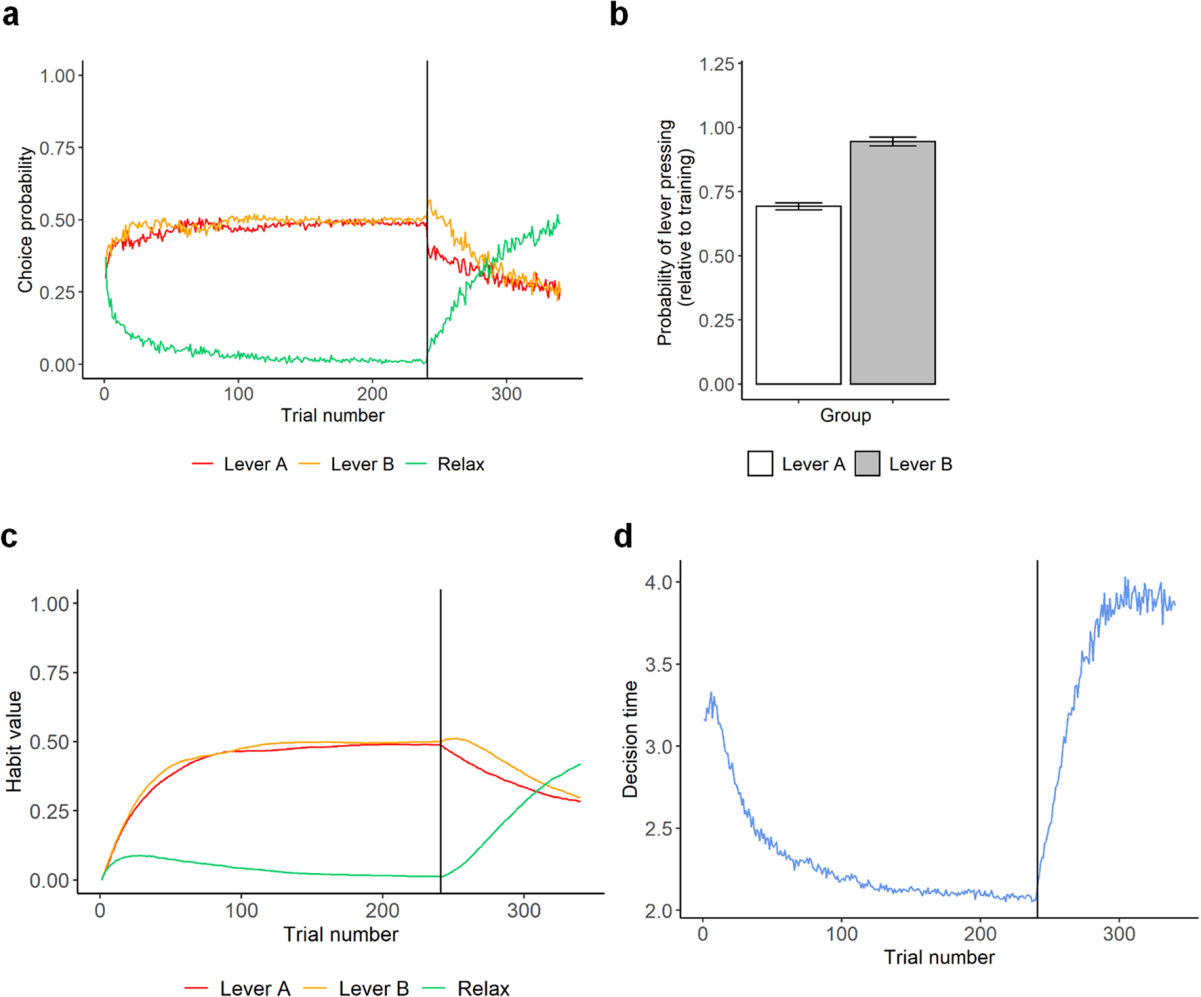
Simulated results for devaluation paradigm with concurrent schedule. **a** Change of choice probability over time; **b** Aggregated response rates (relative to the end of training) after devaluation (first 20 trials used); **c** Habit value; **d** Decision time

As in Fig. 7a and b, results were consistent with the empirical finding: at the point of devaluation, choice probability decreased sharply for the devalued response (lever-press A), while it increased for the non-devalued one (lever-press B). Unlike the classic devaluation experiments, even after extensive training, habit strengths for both responses were only moderate (around 0.5, see Fig. 7c) because of the competition, so the shift in starting points could not compensate for the disadvantages of the devalued response in terms of sampled attribute values. The model also predicted decision time to decrease gradually during training, and to increase greatly in the extinction phase, eventually becoming slower than the decision time at the start of training.

### Study 3: Reversal Learning

Reversal learning refers to learning tasks where payoffs of behavioral responses are occasionally reversed during the task. For example, in Pessiglione et al. (2005), following two stimuli with equal appearance probability, human participants learned in three phases to either press a button (go response) or withdraw from pressing a button (no-go (NG) response) in order to earn as many points as they could. In the training phase, the go-response earns points for one stimulus, while the NG-response earns points for the other. In the reversal phase, the reward-generating stimulus-response mapping was reversed. In the final extinction phase,^8^ the NG-response earns points for both stimuli. The basic finding was that people needed time to gradually learn the changes in the underlying reward probabilities and decision time fluctuated in time: responses became faster when a reward-structure was learned and slower when the structure was reversed.

We used the same task structure as in Pessiglione et al. (2005). Learning agents were assumed to primarily focus on accumulating points (${G}_{point}=0.8$) and to a lesser degree on conserving energy (or to obtain leisure, ${G}_{leisure}=0.1$). Probabilities of obtaining points were either 0 or 1 for the responses depending on the phases (training, reversal, or extinction), while probabilities of obtaining leisure were all set to 1, since the button-pressing responses do not consume much energy for humans. The numbers of trials in the three phases were set to 150, 200, and 150 (as in Keramati et al., 2011). Five-hundred simulations with homogenous agents were run to obtain the results.

**Fig. 8:**
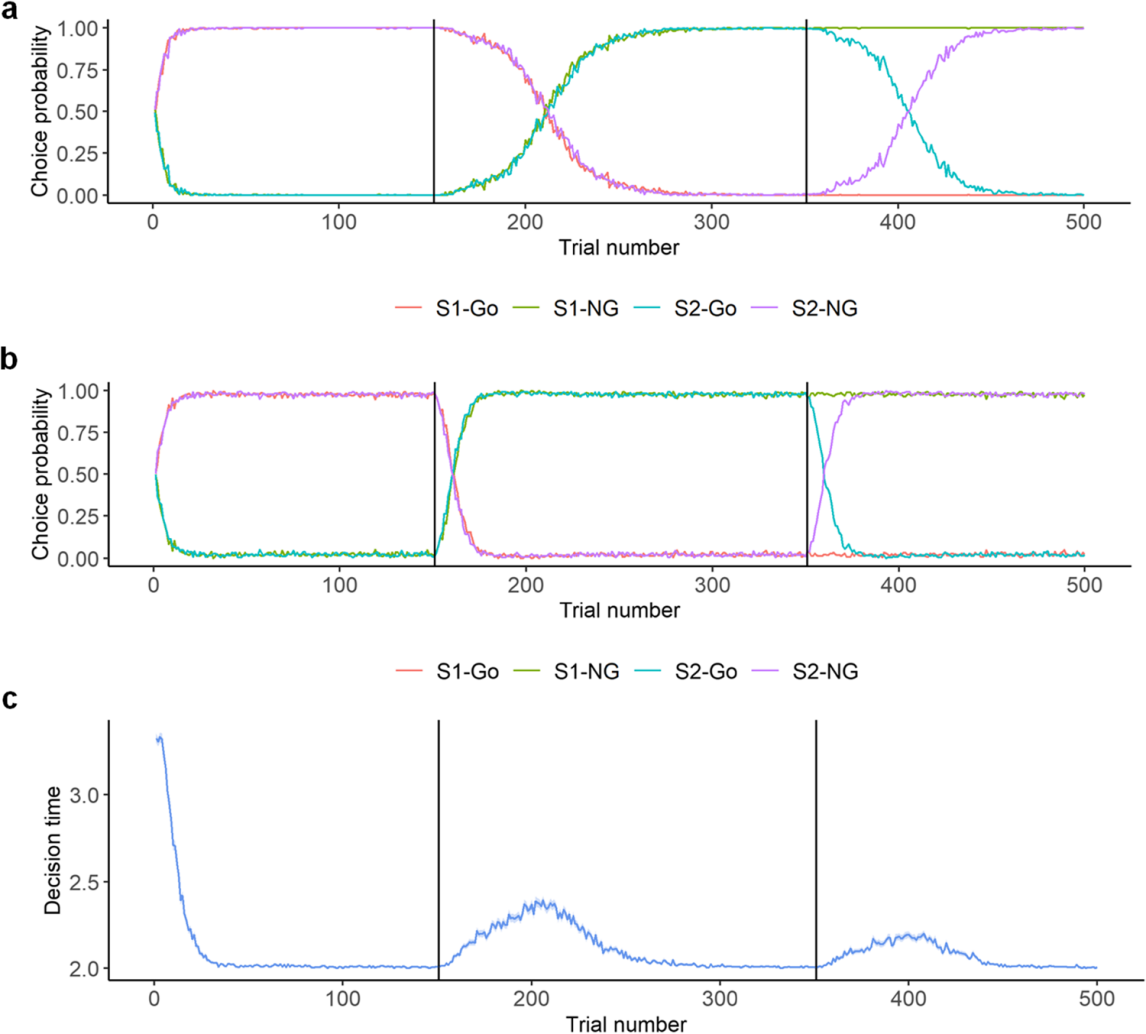
Simulation results of reversal learning. **a** Choice probabilities with ${\alpha }_{H}$ = 0.04; **b** Choice probability with ${\alpha }_{H}$ = 0; **c** Decision time with ${\alpha }_{H}$ = 0.04

Consistent with previous studies (Keramati et al., 2011; Miller et al., 2019), results confirmed that the simulated agents could learn to adapt to changes in reward structure, and indeed the changes of response patterns were gradual rather than steep (Fig. 8a). It should be noted that the habit system or a non-zero ${\alpha }_{H}$ is not essential for producing the basic pattern. Even without habit formation (${\alpha }_{H}$ = 0), the changes in response pattern cannot be completely abrupt, as it takes time to update beliefs about reward probabilities (Fig. 8b). However, it was clear that the changes were much slower with habit formation (in over 100 trials instead of only 30 trials).

Unlike Keramati et al. (2011), our model predicted gradual rather than sudden changes of decision time (Fig. 8c). Consistent with the empirical results (Pessiglione et al., 2005), decision time after the extinction phase increased about 1/2 less than after the extinction phase, because in the extinction phase reversal only applied to one stimuli.

## Parameter Recovery Exercise

So far, we have shown that our sequential sampling model can qualitatively reproduce data patterns found in several empirical studies. A logical next step is to fit the model quantitatively to empirical data with its free parameters to be estimated from the data. Not only can model fitting -provide more rigorous tests of the model, but it can also be used to examine individual differences in key model parameters, as well as the influences of specific experimental manipulations on model parameters. Fitting a complex cognitive model as the one we have developed to data entails two big challenges. First, the complexity of the model makes it very difficult to derive the likelihood function of the model, rendering most traditional (likelihood-based) estimation methods infeasible. Second, given the large number of free parameters, it is questionable to what extent the parameters can be uniquely identified from data as different combinations of parameter values may produce indistinguishable data patterns.

While it is beyond the scope of the paper to fully address these challenges, we evaluated whether and to what extent free model parameters could be estimated through a parameter recovery exercise, i.e., comparing estimated model parameters from simulated data to the true parameter values that were used to generate the data. In the absence of a likelihood function, model fitting was made possible by a technique called the approximate Bayesian computation (ABC; Turner & Van Zandt, 2012), one of several likelihood-free estimation techniques that have been applied to other sequential sampling models (e.g., Miletić et al., 2017; Turner & Van Zandt, 2018; Turner et al., 2018). In short, with ABC, one attempts to approximate the posterior distribution of a model parameter by sampling candidate values from its prior distribution and then evaluating the candidates in terms of whether the model with the candidate values can simulate data that are close enough to their empirical counterpart. If the simulated data and empirical data are close enough – distance between their corresponding summary statistics smaller than a *tolerance* threshold – those candidate value are retained for building the posterior (accepted). Otherwise, they are disregarded (rejected). It has been shown that under certain conditions (e.g., proper distance function, sufficient summary statistics, and small enough tolerance), an ABC-approximated posterior will be equal to the true posterior (Beaumont, 2010). For the exercise, we followed the tutorial paper by Turner and Van Zandt (2012), which provides a thorough and accessible introduction to ABC.

We started by trying to recover only one model parameter, the habit learning rate (${\alpha }_{H}$). This initial step was used to see if ABC would be feasible for our modeling problem and to explore the impact of data type on the estimation performance. For this step, parameter estimation was based on data simulated for a sample of agents’ behaviors in the moderate training with devaluation condition^9^ in a typical outcome-devaluation experiment (as in simulation Study 1). We repeated the parameter estimation procedure in nine different conditions, i.e., with three different true values for ${\alpha }_{H}$ (0.005, 0.04, and 0.2) and three different data types – choice, decision time, and habit value. In each condition, a simple ABC rejection algorithm was used for evaluating candidate values and 1000 accepted candidates were required for forming the posterior distributions (see Supplementary Information for more details about the methods and procedure). Using *R* and the *doParallel* package (Weston & Calaway, 2022), the computation time for each condition was between 1.6 and 4.1 h on Window computers with 9- or 16-core CPUs.

**Fig. 9:**
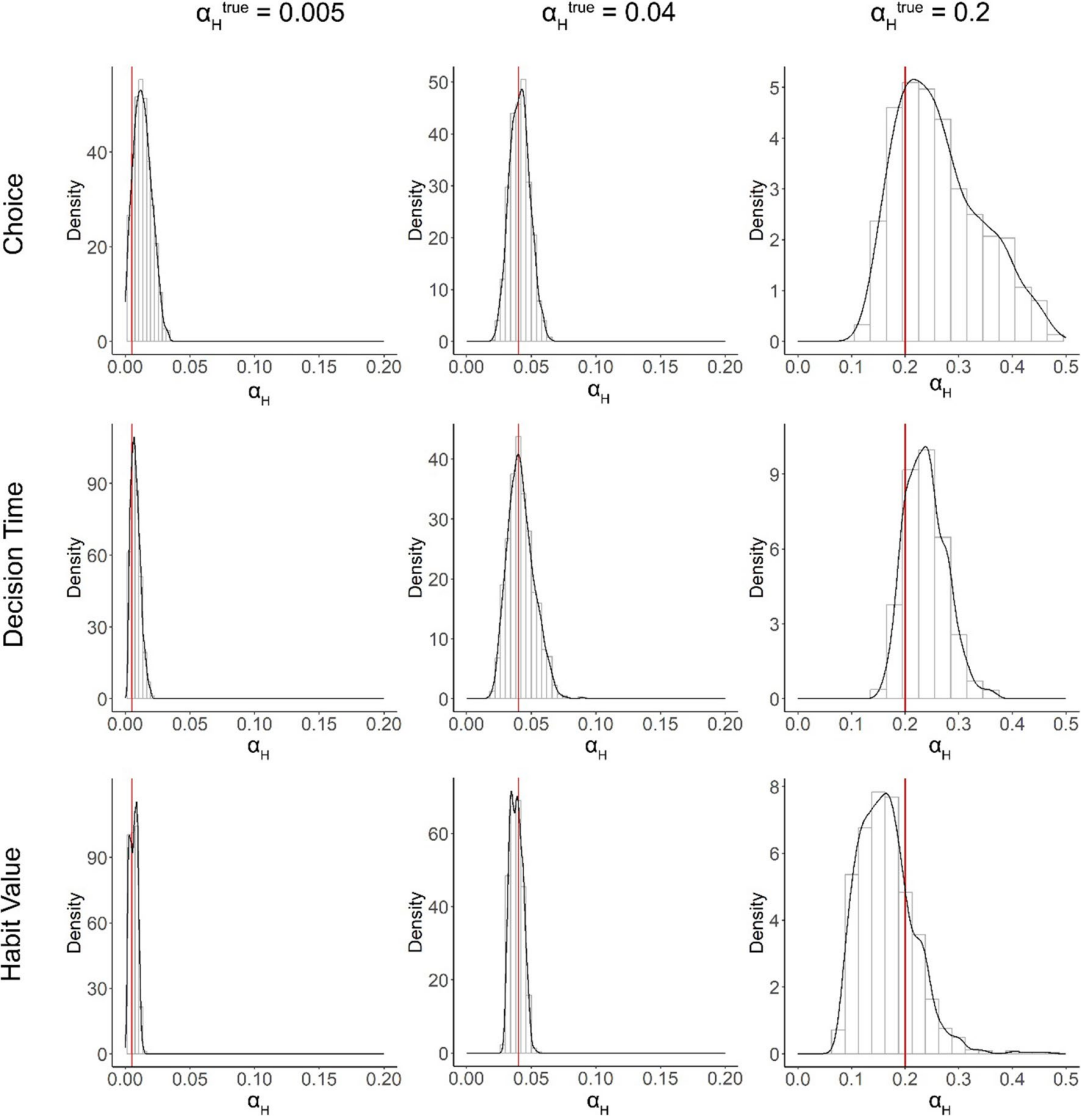
Estimated posteriors for ${\alpha }_{H}$ for the nine parameter recovery conditions using ABC. The read verticals lines indicate the true parameter values for ${\alpha }_{H}$

Figure 9 shows the estimated posteriors of ${\alpha }_{H}$ for the nine conditions. For ${\alpha }_{H}$ = 0.04 (the value used in the simulation studies), results for all three data types were very good, as evidenced by the narrow posterior distributions around the true parameter value and the accurate point estimates (means) and credibility intervals (CI) (*choice*: ${\widehat\alpha}_{H}$ = 0.041, 95% CI = [0.027, 0.057]; *decision time*: ${\widehat\alpha}_{H}$ = 0.042, 95% CI = [0.025, 0.064]; *habit value*: ${\widehat\alpha}_{H}$ = 0.039, 95% CI = [0.031, 0.048]). Parameter recovery was also quite successful for very small ${\alpha }_{H}$ (0.005), although using choice and decision time data resulted in slight overestimation (*choice*: ${\widehat\alpha}_{H}$ = 0.013, 95% CI = [0.002, 0.027]; *decision time*: ${\widehat\alpha}_{H}$ = 0.008, 95% CI = [0.003, 0.016]; *habit value*: ${\widehat\alpha}_H$ = 0.006, 95% CI = [0.002, 0.011]). Performance of ABC for larger ${\alpha }_{H}$ (0.2) was visibly worse, as shown by the much wider posterior distributions. Both choice and decision time data led to overestimation (*choice*: ${\widehat\alpha}_{H}$ = 0.26, 95% CI = [0.14, 0.44]; *decision time*: ${\widehat\alpha}_{H}$ = 0.24, 95% CI = [0.17, 0.32]), while using habit value resulted in underestimation (${\widehat\alpha}_{H}$ = 0.17, 95% CI = [0.09, 0.28]). In general, using habit value data for parameter estimation led to the best results given the close relationship between the variable habit value and the habit learning parameter. However, since habit value is not directly observable in empirical studies, we only used it to demonstrate the capability of ABC algorithms under the most favorable conditions. In contrast, both choice and decision time are variables that can be easily measured in empirical experiments. Therefore, the good results in those conditions suggest the promise of using ABC for estimating model parameters from real empirical data.

We went further to explore the possibility of recovering multiple model parameters at the same time. In addition to $${\alpha }_{H}$$, two more parameters were considered – the uncertainty parameter (γ) in goal-directed learning and the leakage parameter ($${S}_{self})$$ in preference accumulation. The same parameter values as in the simulation studies were used for generating the data for parameter recovery ($${\alpha }_{H}$$ = 0.04, = 0.1, and $${S}_{self}$$ = 0.99). Because the increased dimensionality, a more sophisticated algorithm called ABC population Monte Carlo sampling (ABC PMC) was used to search through the much larger parameter space (see Supplementary Information for detailed procedure; also see Turner & Van Zandt, 2012 for a tutorial). Working in the same computing environment, around 40 h were needed to approximate posterior distributions (1000 candidates) for choice and decision time data respectively.

**Fig. 10:**
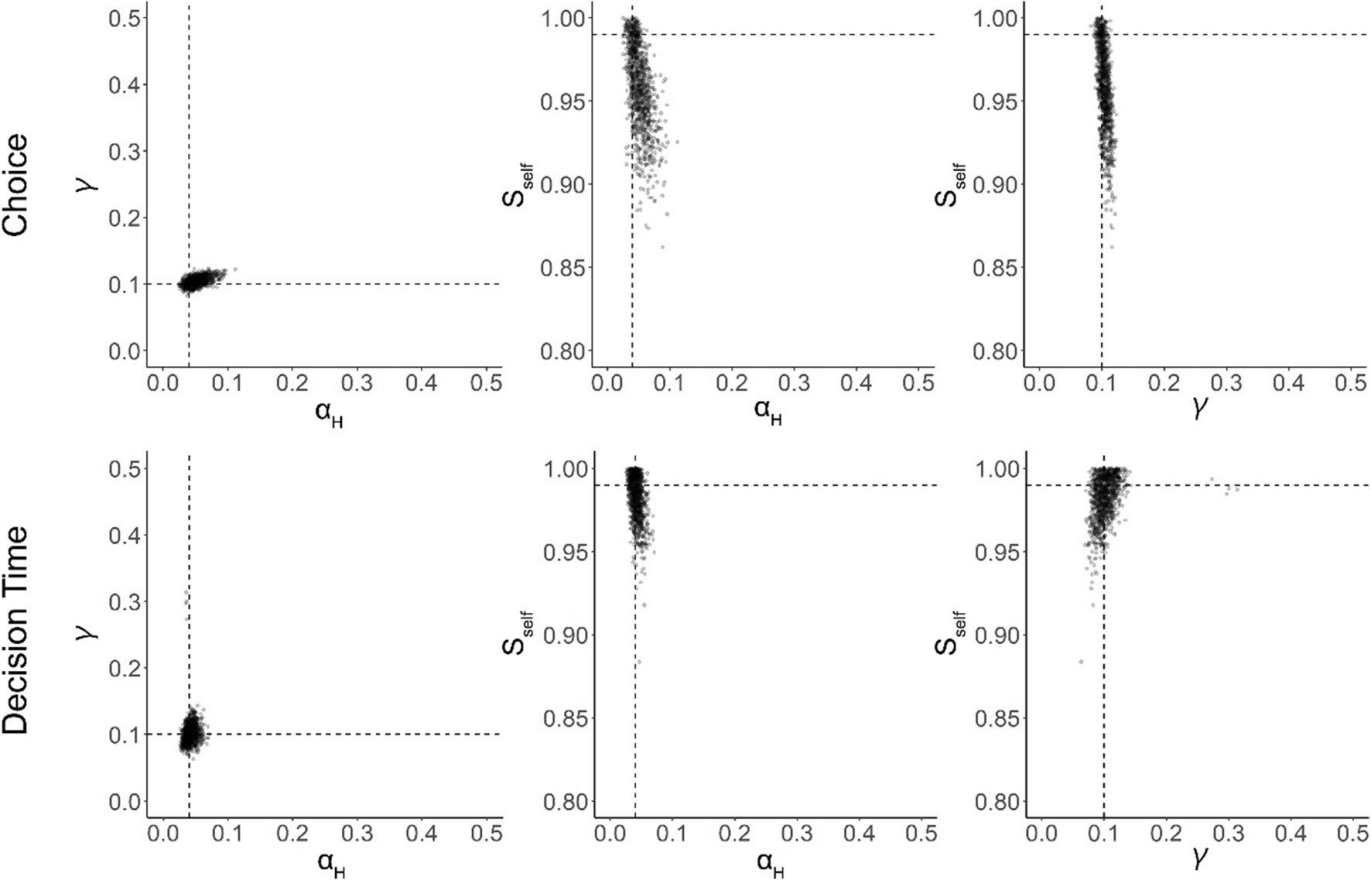
Scatter plots showing the estimated joint distributions for pairs of the three model parameters, habit learning rate (${\alpha }_{H}$), uncertainty parameter (γ) and leakage parameter (${S}_{self}$). The dashed lines indicate the true parameter values used for generating the reference data

Figure 10 shows the estimated joint posterior distributions for each pair of the three parameters in the sequential sampling model. For both choice and decision time data, the recovery of ${\alpha }_{H}$ and γ was very precise. The estimates were unbiased for γ (*choice*: $\widehat{{\upgamma }}$ = 0.10, 95% CI = [0.09, 0.12]; *decision time*: $\widehat{{\upgamma }}$ = 0.10, 95% CI = [0.08, 0.13]) and only a minor overestimation for ${\alpha }_{H}$when estimated from choice data (*choice*: ${\widehat\alpha}_{H}$ = 0.052, 95% CI = [0.031, 0.081]; *decision time*: ${\widehat\alpha}_{H}$ = 0.042, 95% CI = [0.030, 0.059]). For the leakage parameter ${S}_{self}$, the much wider posterior distributions (see Fig. 10, middle and right panels, along the y-axis) suggested less precise estimations (choice: ${\widehat{S}}_{self}$ = 0.96, 95% CI = [0.90, 1.00]; decision time: ${\widehat{S}}_{self}$ = 0.98, 95% CI = [0.95, 1.00]). Still, the simulated data were able to move the uninformative prior between 0.8 and 1 to a much smaller region in the parameter space. Overall, the results suggested that estimating multiple parameters simultaneously did not undermine the performance of ABC in working with our sequential sampling model of habit-goal interaction.

## General Discussion

We have shown that a sequential sampling approach to the integration of habits and goals can reproduce empirical results from three instrumental learning paradigms: classic devaluation, devaluation with a concurrent schedule, and reversal learning. This was achieved by a rather straightforward implementation of the MDFT, with only two additional theoretical assumptions: (1) Starting points of preference accumulation are determined by the habit values of behavioral responses; (2) Attribute sampling probabilities are based on the importance and task-relevance of the corresponding goals. The sensitivity analysis and the fact that the same parameters were used in all three studies speak to the strength of our central theoretical propositions.

### Comments on Effect-Generating Mechanisms

One of the many merits of computational modeling is that it helps researchers to think more deeply about the cognitive mechanisms underlying a behavioral phenomenon (Smaldino, 2017). In a modeling and simulation exercise, very often some theory-based mechanisms are expected and are built into the model on purpose, but other contributing factors to an effect are only discovered after the simulation. In our case, the anticipated central theoretical tenet is that habitual responses are “mistakenly” selected even after outcome devaluation or action-outcome re-mapping because baseline preferences for the habitual options are elevated through past choices. This specific mechanism was speculated by the creator of MDFT (Roe et al., 2001) and is indirectly supported by the research on “choice inertia” in perceptual decisions (Akaishi, et al., 2014; Bode et al., 2012; Mulder et al., 2012; van Ravenzwaaij et al., 2012). While this mechanism proves to be important, two additional mechanisms were necessary for reproducing the effects, especially the insensitivity to outcome devaluation after extensive training.

First, the unattainable goals in a task environment turned out to be important. Even though the preference signal for the habitual response is elevated to be close to the decision threshold at baseline, it still needs additional uplifts to go over the threshold. After outcome devaluation, the primary goal (e.g., obtaining food) is rarely sampled and the remaining “attainable” goal (e.g., leisure) is incompatible with the habitual response (e.g., lever-pressing). It is the occasional sampling of the unattainable goals that pushes the elevated preference signal to reach the threshold. Intuitively, when an agent is deprived of its primary goal in a task environment, the agent starts to explore other goals (albeit unrealistic ones), which accidentally trigger habitual responses.

Second, the uncertainty-injection parameter γ in goal-directed learning moderates the extent to which the unattainable goals contribute to the habitual responses. Sensitivity analysis shows that some uncertainty injection (0.05 < γ < 0.15) is needed for an agent to maintain some associations between the habitual response (e.g., lever-pressing) and the potential satisfactions of the unattainable goals. This can be considered adaptive if the response-outcome or action-reward mappings in the agent’s environment are expected to change over time. We suspect that the γ parameter may also provide an explanation why it is hard to replicate the training-dependent outcome devaluation effect in humans (see de Wit et al., 2018). While human studies are designed to emulate the paradigm of outcome devaluation in rodents, it is reasonable to assume that human participants have considerably lower γ than rodents in their task environments. For human participants, they should understand that the reward structure (i.e., response-outcome mappings) in a controlled laboratory experiment is unlikely to change dramatically. For example, in an experiment where they press keys to obtain sugary drinks, pressing the keys won’t reward any of their personal goals outside the context of the experiment. In contrast, rodents are likely to lack this knowledge and treat the experiment environment (their feeding cage) as the “real-world” where they live in. Our sensitivity analysis indeed suggests that with lower values of γ (< 0.05), the model produces data patterns that are more similar to those in the human studies (de Wit et al., 2018).

Third, it is worth mentioning that one modification to the original MDFT is required to reproduce the outcome devaluation effect, i.e., the removal of the contrast matrix $\mathbf{C}$ in calculating valence or drift rate. The inclusion of the contrast matrix in MDFT implies that valence measures the relative advantages and disadvantages of different responses considering their attribute values for the sampled attribute, rather than their own attribute values. There is currently no consensus in the field on whether preference signals (accumulators) should represent the competitive advantages/disadvantages among responses (relative accumulators) or the independent attribute values of the responses (absolute accumulators). For example, this contrast matrix is not used in other sequential sampling models of value-based decision-making, such as the associative accumulation model (Bhatia, 2013), the multiattribute linear accumulator ballistic model (Trueblood et al., 2014), and the leaky, competing accumulator model (Usher & McClelland, 2001). Even without the contrast matrix, competitions among responses options are still captured by the lateral inhibition in the temporal preference accumulation in our model and in the models above. We found that the inclusion of the contrast matrix severely attenuated the impact of habit strength on the sequential sampling process, thus obliterating the outcome devaluation effect.

### Relations to Other Computational Models

Our work provides a theoretically plausible alternative to arbitration models (Daw et al., 2005; Keramati et al., 2011; Miller et al., 2019; Pezzulo et al., 2013). Instead of competing with each other through centralized arbitration, habits and goals may be integrated dynamically to produce behavioral responses. Sometimes, habits and goals are congruent, so they jointly push responses in the same direction (e.g., the start of any learning process). In other cases, habit-goal conflicts emerge from the same process, when the goal-related attribute values become incongruent with the habit values obtained from prior behavior repetitions, for example, after goal devaluation or reward structure reversal. It remains plausible that habit values and goal-related attribute values are learned in distinct neural systems (Yin & Knowlton, 2006), but at decision moments both value signals are integrated into a single decision-making circuit. This hypothesis should be evaluated in future neurophysiological research, preferably combining existing insights about the neural underpinning of learning (e.g., Dolan & Dayan, 2013; Yin & Knowlton, 2006) and of decision-making (e.g., Dunovan & Verstynen, 2016; Kable & Glimcher, 2009; Rangel et al., 2008; Shadlen & Shohamy, 2016).

Given that quantitative model comparison is beyond the scope of the current paper, the feasibility of our approach does not lend itself to being superior to the arbitration models. It should be also noted that arbitration models do not necessarily implement a “winner-takes-all” approach to response selection. For several models in the literature, a “weighted-average” approach can be taken so that inputs from the habitual and goal-directed systems are weighted by the arbitrator to influence response selection (e.g., Miller et al., 2019; Pezzulo et al., 2013). One can argue that this approach also “integrates” habits and goals. What distinguishes our model from the abstraction model is that it replaces the softmax function with a cognitive process model, i.e., sequential sampling and preference accumulation. If one only looks at responses or choices, we expect the “weighted-average” arbitration models to be able to approximate the behaviors of our process model, thus making it difficult to compare their verisimilitudes based on choice data alone. However, only our process model can make theory-based predictions about decision time, which has been recognized as crucial for demonstrating habits in humans (e.g., Hardwick et al., 2019; Luque et al., 2020).

Our model shares two theoretical stances with Miller et al. (2019). First, both models separate goals values from goal-related attribute values, even though goal values as static decision weights in their model rather than the dynamic precursors of attribute sampling probabilities as in ours. This separation implies a double disassociation that devaluation only depletes goal values, while extinction test only affects goal-related attribute values. In contrast, other models implement both devaluation and extinction as changes to reward probabilities or directly to state-action values (Daw et al., 2005; Keramati et al., 2011). We believe that a separation is theoretically favorable, as it has been made in other theoretical frameworks (e.g., as *outcome value* and *outcome contingency* in learning theories, and as *decision weight* and *attribute value* in decision-making models), and there is evidence that they have distinct neural substrates (Kable & Glimcher, 2009; Rangel et al., 2008). Second, our work adds to Miller et al. (2019) that for explaining classic findings in instrumental learning, a value-free view of habit (Miller et al., 2018; Pauli et al., 2018) is at least as effective as the previous value-based view of habit (Dolan & Dayan, 2013). Our work cannot directly evaluate the verisimilitudes of the two views, but the assumption of mapping habit values to starting points in sequential sampling models is more consistent with Hebbian learning algorithms (value-free) than with model-free reinforcement learning algorithms (value-based) of habit learning (see Akaishi et al., 2014).

We are not the first to combine reinforcement learning and sequential sampling models. As reviewed in the introduction, several researchers have explored this idea and showed that using a drift diffusion model as the response selection model in reinforcement learning can explain choice and decision time data from a human decision-making task with reward feedback (i.e., a bandit task) (Fontanesi et al., 2019; Frank et al., 2015; Pedersen et al., 2017). Still, we are the first to implement a separate habit learning component in the overall model and to examine the interaction between two learning systems – habit and goal-directed learning. An obvious difference is the use of drift diffusion model in earlier works versus a modified MDFT in our work. Our choice was motivated by the wide application of MDFT in value-based decision-making, especially its superiority in accounting for context effects in multialternative multiattribute choices (Berkowitsch et al., 2014; Hotaling & Rieskamp, 2019). Given the many similarities between the two models, one can expect the earlier models (the so-called reinforcement learning drift diffusion models or RLDDM) may also be able to reproduce our findings if habit strength is modeled in RLDDM in the same way.

### Theoretical Implications for Habit Research and Value-based Decision-making

A major controversy in habit research is the debate over the relationship between habits and goals, or whether habitual behaviors are goal-dependent or goal-independent (Gardner & Lally, 2022; Kruglanski & Szumowska, 2020; Marien et al., 2019; Wood et al., 2022). There is no doubt that habits originate from instrumental learning where the repeated behaviors serve to satisfy the goals of an organism. However, there is not much consensus beyond this point. We believe that a general distinction between goal-dependence and goal-independence is not useful and researchers need to ask a more nuanced question – in which part of the cognitive and behavioral processes of motivated actions are habits dependent on or independent from goals?

In terms of learning processes, both the traditional and the current dominant views see habit learning and goal-directed learning as two distinct systems. As early as in Thorndike’s time, a distinction between stimulus-response association (S-R) and action-outcome association (A-O) was made, as well a distinction between “law of exercise” and “law of effect” (Thorndike, 1932). In the current view in neuroscience, two distinct systems exist and different brain regions are believed to underlie habit learning (e.g., dorsolateral striatum) and goal-directed learning (e.g., dorsomedial striatum) (Dolan & Dayan, 2013; Yin & Knowlton, 2006). It should be noted that the traditional distinction was blurred to some extent since Daw et al. (2005)’s seminal work on modeling habit learning as model-free reinforcement learning that depends on goal-related action outcomes, but still the two learning systems are treated as largely separate or independent (but see Daw et al., 2011; Gläscher et al., 2010).

In terms of decision-making or the control of behavior, one can also ask the question when a behavior is habitual, whether goal-related constructs (e.g., attitude and intention) still influence behavior. Although many researchers may not believe in fully automatic behaviors, they sometimes define habits as such. For instance, Wood and Neal (2009) described habits as “a type of automaticity characterized by a rigid contextual cuing of behavior that does not depend on people’s goals and intentions” (p. 56). However, both empirical studies in controlled environments and causal observations of real-life habits suggest that in the absence of the original goal (e.g., devalued by an experimental manipulation), even highly habitual behavior will gradually disappear, even though more intensive training leads to slower extinction (Adams, 1982; Dickinson, 1985; Tricomi et al., 2009).

These formal and informal observations are not at odds with most arbitration models. If an “weighted-average” mechanism is used, then clearly after arbitration inputs from both systems are “integrated” to influence behavior, even though the underlying cognitive process is not specified. Even when a “winner-takes-all” approach is employed, abstraction models will simulate decaying habits after goal devaluation if habit learning is modeled as a form of response-outcome learning (e.g., model-free reinforcement learning, Daw et al., 2005). In line with these models, our sequential sampling approach predicates a precise form of habit-goal integration at all times. Even when a habit is very strong (starting point close to the decision threshold), still goal-related attribute values can influence the accumulation process, albeit to a very limited extent. The attenuated impact of goals is consistent with the group-level habit-intention or habit-attitude interaction effect found in applied health psychology research, i.e., strong habits attenuate the influence of intention and attitude on behavior (e.g., Triandis, 1977; Verplanken et al., 1994; Zhang et al., 2022a, b; for reviews, see Gardner, 2015; Gardner et al., 2020).

Our model also has implications for the role of uncertainty and speed-accuracy trade-offs in value-based decision-making. Conceptualizations of uncertainty and speed-accuracy trade-offs have been made in earlier models (Daw et al., 2005; Keramati et al., 2011; Kool et al., 2017), but uncertainty was computed as a higher-order mathematical property, such as variance of distributions. Rather, uncertainty is realized in our model as the sampling of values from distributions in a stochastic process of preference accumulation. In addition, speed-accuracy trade-offs are naturally incorporated in any sequential sampling model (e.g., Ratcliff & Rouder, 1998), as more accumulation steps reduce uncertainty but lead to longer decision times.

Finally, the idea of sampling values from distributions for decision-making coincides with the Thompson sampling approach of solving repeated decision problems (Bandit problems), which usually achieves optimal balance between exploration and exploitation (Russo et al., 2018). Thompson sampling can be seen as a special case of sequential sampling with only one step. In this sense, sequential sampling with more than one step would favor exploitation more than exploration, depending also on the decision threshold. By shifting starting points closer to threshold, strong habits further enhance exploitation. In contrast, unattainable attributes in our model provide a mechanism against over-exploitation, since the under-explored responses tend to have higher mean expected values for those attributes (Fig. 5f). In the events of sudden environmental changes (e.g., devaluation of primary goals), this mechanism counteracts habits to promote exploration. Future research should examine the role of habits in the exploration-exploitation dilemma and in reverse the role of the dilemma in instrumental learning.

### Limitations, Applications, and Future Work

One strength of the sequential sampling approach is its ability to predict decision time. We have exploited this strength only to a limited extent, for example, in producing the temporal change of decision time that closely matches the one in the reversal learning experiment, but much more can be done in future work. This strength has become even more valuable as recent studies pointed to the importance of examining decision time in studying human habits (Hardwick et al., 2019; Luque et al., 2020). For instance, Hardwick et al. (2019) argued strong habits (cue-behavior mappings) might only trigger the preparation of a response, but not necessarily its initiation and execution. Habitual preparations are often overridden by goal-directed control, but can be unmasked by forcing people to respond at a faster pace. Our computation model can be considered as a formalization of their proposal – habitual response preparations can be represented by the elevated baseline preferences in preference accumulation, and the forced fast decisions makes it more likely that the preference signal for the habitual response is still higher than the one for the non-habitual but correct response at the moment of committing to a decision.

A particularly interesting prediction from our model is that decision time for the habitual response after outcome devaluation will also increase. The reason is that it takes more time for the habitual preference signal to reach the threshold when the positive drifts only come from the sampling of unattainable goals rather than the devalued primary goal. Intuitively, this means that when people mistakenly respond in a habitual way, these “slips of actions” are still slower than their counterparts before the devaluation. In Luque et al. (2020), the authors used the response time switch cost as a measure of habit, but they looked at the time cost of switching from a habitual response before outcome devaluation to the correct and non-habitual response after devaluation. The prediction that habitual responses also become slower after devaluation has not been examined. This prediction is “risky” and would be “surprising” in the absence of our model (e.g., not predicted by Keramati et al., 2011), so it provides a strong test for our model in future research (Meehl, 1990; Roberts & Pashler, 2000).

While our current contributions primarily concern model development and simulation, we also performed a small-scale parameter recovery exercise using ABC algorithms. The preliminary findings suggest that fitting our model to data to estimate model parameters is feasible at least in principle. Still, several limitations and challenges need to be considered before interfacing the model with real empirical data. First, our exercise was limited to the estimation of three parameters, while the full model contains many more potential free parameters. Given that the recovery of only three parameters took more than one day, computation time is a concern. However, computation time will be less of an issue over time and it can be substantially reduced by using more computing resources and/or by implementing the algorithm in faster programming languages than *R*. Another strategy would be to constrain some parameters (e.g., scaling parameters and parameters that are strongly constrained by theories) and leave only the parameters that relate to meaningful individual differences to be estimated (e.g., ${\alpha }_{H}, \gamma$, ${S}_{self}, {S}_{other}$, and individual differences in task-specific goal values). Second, given the stochastic nature of the model and the true underlying processes, relatively large sample sizes (e.g., at least 50 to 100; see Supplementary Information for the exact number used) are required for obtaining good results using ABC. In our exercise, those 50 or 100 simulated agents had the exact same cognitive parameters, but empirically, individual differences exist for most of the cognitive parameters. Thus, an ABC-version of the popular hierarchical Bayesian modeling will be required (see Turner & Van Zandt, 2012). Finally, working with empirical data will mean much less correspondence between the measured variables and the simulated quantities by the cognitive model. For example, while the model predicts pure decision time in terms of accumulation steps, measured decision time in behavioral experiments is much noisier and reflects more than just decision-making processes.

Despite the remaining challenges, the demonstrated possibility of parameter estimation is particularly important for the practical value of our model. One of the greatest challenges in applied habit research is to reliably measure individual differences in how fast people form and break habits. While habit strength has been measured by self-reports to estimate the speeds of habit formation and decay in the real-world (e.g., Lally et al., 2010), the usefulness of the results is bounded by the validity of the scale and the general limitations of self-report (see de Wit et al., 2018). Our modeling approach provides an attractive alternative: individual differences in habit growth and decay parameters^10^ can be studied by fitting our model to human choice and decision time data obtained from various instrumental learning experiments. The estimated individual differences can then be used in many ways, including predicting habit formation in the real-world, comparing different healthy and clinical populations, and informing strategies for changing habits.

## Conclusion

In summary, our work has demonstrated the potential of considering sequential sampling as a key cognitive mechanism underlying habit-goal interactions. Because sequential sampling models are well-suited for modeling value-based decision-making, they can help researchers to better connect basic instrumental learning research to human habits in real-world contexts (see Marien et al., 2019) and to study individual differences through model fitting. More broadly, our work extends an emerging research line of applying sequential sampling models to human reinforcement learning, and encourages a more unified approach to learning and decision-making theories in psychological science.

## Electronic Supplementary Material

Below is the link to the electronic supplementary material.

Supplementary Material 1

## Data Availability

Data sharing not applicable to this article as no empirical datasets were generated or analyzed during the current study.

## Footnotes

1 R code for the model, simulation studies, and parameter recovery exercise can be found in the Open Science Framework (OSF) repository: https://osf.io/ycqdj/. Data sharing is not applicable to this article as no empirical datasets were generated or analyzed during the reported studies.

2 There is an ongoing debate on whether habit learning depends on the decisions alone (value-free, see e.g., Miller et al., 2018, 2019; Pauli et al., 2018) or also on decision outcomes (e.g., value-based, see Daw et al., 2005; Keramati et al., 2011). Because our objective was to propose a new model of habit-goal integration in response selection, we took the value-free view of habit learning for its simplicity and its similarity to the updating rule of prior choice’s effect in perceptual decision-making (Akaishi et al., 2014). In theory, our sequential sampling approach should remain effective under the alternative view of habit learning.

3 For our applications, we assumed that decision-making processes are terminated internally by a decision threshold. However, decision-making processes can also be forced to terminate at a time *t*, and the response with the highest preference at that time is chosen.

4 A different cognitive mechanism with the same consequence is that the agent starts to accumulate preferences for habitual responses earlier than other responses (see also Psarra, 2016).

5 In the original MDFT, valence is computed as ${\mathbf{V}}_{\left(t\right)}=\mathbf{C}{\mathbf{M}}_{\left(t\right)}{\mathbf{W}}_{\left(t\right)}$, where $\mathbf{C}$ is an *N*-by-*N* contrast matrix with all the elements on the main diagonal equal to 1 and all other elements equal to $-1/(N-1)$. We comment on this change we made in the Discussion section.

6 The uppercase *T* in the equation denotes time point or decision point (e.g., trial number in experiments), which is different from the time step *t* in the sequential sampling of each decision.

7 The exact reward probability for food was not decisive for reproducing the devaluation effect, as long as it was high enough so that the full acquisition of the lever-pressing response was achieved.

8 To avoid confusion, it is important to note that extinction phase in Pessiglione et al. (2005) does not mean “no reward” as in other animal learning experiments, but only implies that the active go-response (button-pressing) is unlearned.

9 Given the time constraint, we only obtained full results this condition, but quick explorations suggested that using any of the other three conditions were unlikely to substantially change our main results.

10 While our model currently uses the habit learning equation from Miller et al. (2019), which has a single parameter for habit growth and decay, other models of habit change use two separate parameters, thus allowing different speeds in forming and breaking habits (e.g., Klein et al., 2011).

## Code Availability

R code for the model and simulation studies can be found in the Open Science Framework (OSF) repository: https://osf.io/ycqdj/.
