# Correction: A Sequential Sampling Approach to the Integration of Habits and Goals

**DOI:** 10.1007/s42113-024-00201-z

**Published:** 2024-03-12

**Authors:** Chao Zhang, Arlette van Wissen, Ron Dotsch, Daniël Lakens, Wijnand A. IJsselsteijn

**Affiliations:** 1 Human-Technology Interaction Group, Eindhoven University of Technology, PO Box 513, 5600 MB Eindhoven, The Netherlands; 2 Digital Engagement, Cognition and Behavior Group, Philips Research, Eindhoven, The Netherlands; 3 Present Address: Amsterdam, Netherlands

**Correction: Computational Brain & Behavior**

10.1007/s42113-024-00199-4

The original online version of this article was revised to correct the affiliation of coauthor Ron Dotsch. The correct affiliation is: Current address: Amsterdam, Netherlands.

The author note for this article was also corrected to read:

Ron Dotsch was employed at Philips Research when he contributed to the reported research.

The original article has been corrected.

