# Supplementary material for: A Sequential Sampling Approach to the Integration of Habits and Goals
Source: Comput Brain Behav. 2024 Mar 5;7(3):480–501. doi: 10.1007/s42113-024-00199-4 (PMC13298635; doi:10.1007/s42113-024-00199-4)
Supplement: Supplementary file 1 — Supplementary Material 1 [file 42113_2024_199_MOESM1_ESM.pdf]

Supplementary Information: Detailed methods and procedures of the parameter recovery exercise

Methods and procedure for estimating habit learning rate

The general procedure for recovering habit learning rate (α_H) is as follows. With a target value for α_H and fixed values for all other parameters (i.e., values also used in all simulation studies), we used the model to simulate a fixed number of agents' behaviors in one condition of the outcome-devaluation experiment as in Study 1 (e.g., 40 training trials and 100 extinction trials as in the moderation training conditions). The simulated data were then used to derive the aggregated temporal patterns of choice (response selection), decision time, and habit value for all the agents over the 140 trials. The three variables could be seen as three different types of data for parameter estimation. Both choice and decisions can be easily measured in experiments so they can be used for parameter estimation based on empirical data. Habit value is not directly observable but we used it as a baseline condition where ABC algorithms have the highest chance of success given the close relationship between the variable and α_H.

A simple ABC rejection algorithm (see Turner & Van Zandt, 2012) was then used for obtaining the posterior of α_H based on the reference data. From a uniform distribution between 0 and 0.5 (i.e., the prior), candidate values for α_H were drawn and then used with the model to simulate data to evaluate how close they resembled the reference data. For computation efficiency, a smaller number of agents (e.g., 10) were simulated in the same task environment and aggregated temporal patterns of choice and decision time were derived to predict the reference patterns. Mean absolute error (MAE) of prediction was computed as the model prediction error (see Figure A1 for an example of correlation between parameter candidate value and MAE). If the error was smaller than

a tolerance parameter, the candidate value was accepted. The process continued until a large number of candidates (e.g., 1000) were accepted to form the posterior distribution. The specific hyperparameters (i.e., parameters that controlled the ABC) for the three data types are shown in Table A1. The different tolerance thresholds were chosen to have similar levels of acceptance rate (1-2%) for the different conditions.

Figure A1. Correlation between candidate value of habit learning rate and MAE (from the choice data conditions). The dashed line shows the level of tolerance.

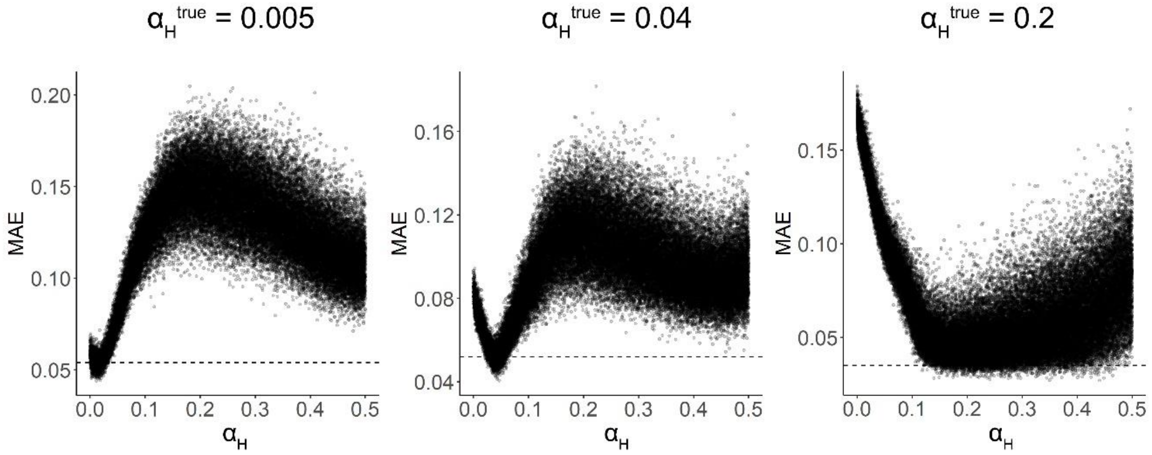

Table A1. Hyperparameter used for the parameter recovery of α_H using ABC.

Data type	N_{accept}	N_{agent} (reference data)	N_{agent} (simulation data)	Tolerance
Choice	1000	100	50	0.054 (α_H= 0.005)<br>0.052 (α_H = 0.04)<br>0.035 (α_H = 0.2)
Decision time	1000	50	10	0.35 (α_H = 0.005)<br>0.28 (α_H = 0.04)<br>0.16 (α_H = 0.2)
Habit value	1000	50	10	0.08 (α_H = 0.005)<br>0.03 (α_H = 0.04)<br>0.04 (α_H = 0.2)

Methods and procedure for estimating three parameters at the same time

As with the parameter recovery for a single parameter, we first used the model with the true values for the three parameters (α_H = 0.04, γ = 0.1, and S_{self} = 0.99) to simulate 100 homogenous agents' behaviors in the moderate training with devaluation condition in a typical outcome devaluation experiment. Their aggregated temporal patterns for choice and decision time were summarized for later use.

Because of the increased dimensionality, we used a more sophisticated algorithm called the ABC population Monte Carlo sampling (ABC PMC) to deal with the much larger parameter space. Unlike the simple ABC rejection algorithm, ABC PMC sampling approximates the posterior distributions of parameters through several iterations, through which the search shifts its focus gradually to the more plausible regions for acceptable candidates. This is achieved through two additional mechanisms. First, while in the one first iteration candidates (also called particles in AMC PMC sampling) are sampled from the priors with equal probabilities, in subsequent iterations the population of particles (e.g., 1000) obtained from the last iteration were used to sampling new particles and their probabilities of being sampled (weights) were updated over the iterations. Second, when a particle is sampled, a transition kernel (e.g., a Gaussian kernel) is used to perturbate the sampled value to have a new particle for evaluation (the evaluation part is the same as with the ABC rejection algorithm). The transition kernel is adaptive because the amount of perturbation is determined by the variance of the particle population from the last round. This implies less perturbations when the search is getting closer to the region for the true parameter values. Each iteration is completed when the target number of particles (e.g., 1000) is reached and the population of particles obtained in the final round is used as the posterior

distribution. Finally, it is customary to use a monotonically decreasing set of tolerance thresholds for the iterations, so the acceptance criterion becomes more and more strict. Interested readers can look into our *R* code on OSF and refer to Turner and Van Zandt (2012) for the equations for calculating the weights and kernel variances.

In our specific exercise, we used five iterations with 1000 particles. For perturbation, truncated Gaussian distributions were used in order to find new values around the sampled particles but constraining the values to be within the boundaries of the initial priors for the parameters. Uniform distributions were used as priors for the three parameters (α_H ~ [0, 0.5], γ ~ [0, 0.5], S_{self} ~ [0.8, 1]). For each particle, 50 agents were simulated in order to predict the reference pattern of choice or decision time and mean squared errors (MAEs) were computed for accepting or rejecting the particles. Monotonically decreasing sets of tolerance thresholds were used for choice data ([0.1, 0.08, 0.07, 0.06, 0.051]) and decision time data ([0.35, 0.3, 0.25, 0.2, 0.15]). These thresholds were chosen to calibrate the particle acceptance rate to be around 0.3-0.5%.
